# IL‐34 is a potential biomarker for the treatment of papillary thyroid cancer

**DOI:** 10.1002/jcla.23335

**Published:** 2020-06-23

**Authors:** Ping Zhang, Hao Zhang, Wenwu Dong, Zhihong Wang, Yuan Qin, Changhao Wu, Qi Dong

**Affiliations:** ^1^ Department of General Surgery The First Hospital of China Medical University Shenyang China; ^2^ Department of General Surgery The People's Hospital of China Medical University Shenyang China

**Keywords:** epithelial‐mesenchymal transition, ERK signaling, interleukin‐34, papillary thyroid cancer

## Abstract

**Background:**

Interleukin (IL)‐34 is a recently discovered pro‐inflammatory cytokine and is a vital regulator in different tumor types. However, the function of IL‐34 in thyroid carcinoma has yet to be investigated. In this study, we analyzed the expression of IL‐34 in human papillary thyroid cancer (PTC) samples and determined its effects on the proliferation and apoptosis of PTC cells.

**Methods:**

We examined the expression of IL‐34 in serum and tissue samples of patients with PTC by Western blotting and ELISA assay and analyzed its association with clinicopathological features including tumor size, tumor node metastasis (TNM) stage, and lymph node metastasis (LNM). We selected TPC1 and K1 for knockdown or overexpressing of IL‐34 via small interference RNA transfection. The proliferation of PTC cells was evaluated by CCK8 assay. We further investigated the role of IL‐34 in apoptosis by flow cytometry and studied the protein levels of epithelial‐mesenchymal transition (EMT) biomarkers, phosphorylated extracellular‐regulated kinase (ERK), and total‐ERK (t‐ERK) by Western blotting.

**Results:**

Our results show that IL‐34 is significantly upregulated in serum and tissue samples from patients with PTC. IL‐34 promotes the proliferation and suppresses apoptosis in PTC cells. In addition, IL‐34 can promote the EMT and activate ERK signaling pathway in PTC cells.

**Conclusion:**

This study provides novel evidence that IL‐34 serves as an oncogene in PTC. IL‐34 promotes proliferation, EMT phenotype, and ERK signaling pathway and inhibits apoptosis in PTC cells. Therefore, IL‐34 may be a potent therapeutic target for the treatment of PTC.

## 1 INTRODUCTION

Papillary thyroid cancer (PTC) is one of the most common endocrine malignant tumors, accounting for over 80% of thyroid carcinomas.^1^ Although therapeutic approaches for human PTC including surgery, chemotherapy, and radiation are rapidly with every passing days,^2^, ^3^, ^4^ over 30% of patients present with lymph node metastasis and recurrence within a decade.^5^ Thus, establishing the mechanisms that mediate the invasion and migration of PTC is vital to the treatment of thyroid carcinoma. Multiple molecular signaling pathways have been shown to contribute to the development of thyroid cancer. Among them, the mitogen‐activated protein kinase (MAPK) pathway plays a vital role in the development of thyroid cancer.^6^, ^7^ The ERK pathway is a crucial component of the MAPK signaling pathway, and its activation regulates cell proliferation and differentiation.^8^ Increasing evidence has demonstrated that the ERK signaling pathway is essential for induction of epithelial‐mesenchymal transition (EMT).^9^, ^10^

Interleukin‐34 (IL‐34) is a recently discovered pro‐inflammatory cytokine comprised of 242 amino acids.^11^ It is a ligand of colony stimulating factor 1 receptor (CSF‐1R) and is widely expressed in multiple organs including the brain, liver, heart, and skin. Moreover, IL‐34 can bind to the extracellular domain of CSF‐1R, resulting in phosphorylation of intracellular tyrosine residues, leading to cell proliferation and differentiation.^12^ IL‐34 can stimulate monocyte and macrophage differentiation by secretion of pro‐inflammatory cytokines, such as IL‐6 and TNF‐α.^13^ IL‐34 is involved in diverse autoimmune and inflammatory diseases, and its role as a novel therapeutic target has been proposed. Serum levels of IL‐34 are significantly increased in patients with rheumatoid arthritis (RA) and correlate with disease severity.^14^ In addition, IL‐34 expression is elevated in the serum and intestine of patients with inflammatory bowel disease (IBD).^15^ Several studies have also concentrated on the contribution of IL‐34 in cancer and demonstrated that IL‐34 plays a pro‐tumorigenic role in the tumor microenvironment.^16^ Increased expression of IL‐34 has been found in patients with different kinds of carcinomas, including breast, lung, ovarian, and blood cancer, and it has been reported that its expression is correlated with the progression of tumor metastasis.^17^, ^18^, ^19^, ^20^, ^21^, ^22^ IL‐34 can be induced by cancer cells, which is believed to promote chemoresistance.^20^ Accumulating evidence demonstrates that IL‐34 also plays an important role in tumorigenesis based on its effect on promoting endothelial cell proliferation and transportation of macrophages into tumor cells.^18^, ^23^ For example, IL‐34 promotes osteoclastogenesis and participates in giant cell tumor development of bone.^17^ In hepatocellular carcinoma, IL‐34 exerts its effect by stimulating the differentiation of tumor‐associated macrophages (TAM), leading to tumor metastasis.^24^

Whether IL‐34 can activate ERK signaling and therefore influence PTC has not been evaluated. Therefore, in this study we examined IL‐34 expression in human PTC samples and cell lines, and studied its effect on cell proliferation and apoptosis. We also studied the effects of IL‐34 on epithelial‐mesenchymal transition (EMT) and ERK signaling in PTC cells.

## 2 MATERIALS AND METHODS

### 2.1 Tissue specimen acquisition

This study was conducted with the approval of the ethics committee of First Affiliated Hospital of China Medical University. Informed consent was obtained from all patients with PTC and corresponding controls whose tissue specimens and serum samples were used in this study. All participants provided detailed medical history documentation and underwent a complete physical examination. None of the patients had ever received antithyroid drugs. Subjects with diabetes mellitus or a family history of diabetes, other autoimmune disease, infectious disease, or cancer were excluded. The control subjects with no history of any thyroid disease, confirmed by clinical, hormonal, thyroid ultrasound scan, and the presence of thyroid autoantibodies were included in this study. All patients had a clinical duration of less than 3 years and had been admitted to the hospital for standard thyroidectomies. The fresh PTC tissues were instantly placed in liquid nitrogen and stored at −80°C until further use. The serum samples from each patient and corresponding controls were also obtained and stored until use.

### 2.2 Serum IL‐34 concentrations

Serum IL‐34 levels were analyzed by ELISA assay (R&D Systems, Minneapolis, MN, USA). In general, plates coated with IL‐34 antibody were cultured with fivefold dilutions of serum at room temperature for 2 hours. The plates were washed and further incubated for 2 hours with horseradish peroxidase‐conjugated IL‐34 antibody. The plates were then washed, treated with tetramethylbenzidine, and incubated for 30 minutes. Ultimately, sulfuric acid was added to end the reaction. The absorbance at 450 nm was then detected.

### 2.3 Cell culture and transfection

Human papillary thyroid cancer cell lines including TPC‐1and K1 were purchased from the American Type Culture Collection (ATCC). Cells were cultured in Dulbecco's modified Eagle's medium (DMEM, Gibco) supplemented with 10% fetal bovine serum (FBS, Hyclone), 100 U/mL penicillin (Gibco), and 100 g/mL streptomycin (Gibco). All cells were cultured under a 5% CO_2_ atmosphere at 37°C. The medium was changed once every 2 days, and cells were serially passaged when the cell density reached 70%‐80% confluence. The sample sizes of TPC‐1 and K1 were three. Small interfering RNA targeting IL‐34 (si‐IL‐34) and the negative control (si‐NC) were purchased from Shanghai GeneChem Company. TPC and K1 cell lines were transfected with si‐IL‐34 or si‐NC using Lipofectamine 2000 (Invitrogen) strictly following the instructions.

### 2.4 Lentivirus production and cell transduction

293T cells were transfected with pEZ‐lv105 vector (GeneCopoeia) using Lipofectamine 2000 (Invitrogen). Viral particles were harvested at 72 hours time points following transfection, and viral titers were determined. For viral transduction, 1 × 10^5^ cells were transduced with 1 × 10^6^ recombinant lentivirus‐transducing units in the presence of 6 µg/mL polybrene.

### 2.5 Cell proliferation assay

Cell proliferation was examined using the Cell Counting Kit 8 (CCK8) assay. In brief, PTC‐1 and K1 cells were seeded at 8 × 10^3^ cells/well in 96‐well plates. After culture for 4 days at 24‐hour intervals, the cells were treated with 10 μL of a CCK‐8 reagent (Dojindo, Kumamoto, Japan) for 1 hour. The optical density (OD) was measured at 490 nm using a spectrophotometer.

### 2.6 Flow cytometry analysis of apoptosis

The rates of apoptosis for TPC‐1 and K1 cells were analyzed using Annexin V and PI Detection Kit (BD Pharmingen, San Jose, CA) according to the manufacturer's instructions. Samples were then analyzed with a BD FACS Array (Becton‐Dickinson) using CellQuest software.

### 2.7 Western blot analysis

Proteins from PTC tissues and cells were homogenized in cold PBS containing 0.05% Triton X‐100 and protease inhibitor cocktail (Sigma‐Aldrich). Protein samples were electrophoresed on 10% sodium dodecyl sulfate‐polyacrylamide gels (Sigma), transferred onto PVDF membranes according to standard protocols, and then blocked with 5% dried skimmed milk in TBST for at least 1 hour. The membranes were incubated overnight at 4°C with the following antibodies: anti‐E‐cadherin, anti‐N‐cadherin (1:1000, Abcam), anti‐vimentin, anti‐p‐ERK, anti‐ERK (1:1000, Cell Signaling Technologies), and anti‐GAPDH (1:1000, Abcam) and then incubated with horseradish peroxidase‐conjugated secondary antibodies (1:1000, Abcam) at room temperature for 1 hour after washing 3 times using TBST. Blots were washed 3 times again and developed using an enhanced chemiluminescence kit (Amersham Pharmacia Biotech). Immunoblot band quantification was calculated by means of a Bio‐Rad calibrated densitometer (GS‐800) using the vendor's software (Bio‐Rad Laboratories); GAPDH was used as an internal reference for analyses.

### 2.8 Quantitative real‐time PCR analysis

Total RNA was extracted from placental samples using TRIzol (Thermo Fisher Scientific Inc). Reverse transcription was achieved using Takara Kit (TaKaRa Biotechnology Co., Ltd.). cDNA amplification was then performed using SYBR Premix Ex Taq (TaKaRa) in a Roche 480 Light Cycler. PCR was carried out for 40 cycles according to the following procedure: 95°C for 30 seconds, annealing for 30 seconds, and 60°C for 34 seconds. Each sample was analyzed in duplicate. Relative mRNA expression levels of the target genes were calculated using the 2^−ΔΔCt^ method after normalization to GAPDH. The mRNA sequences used for PCR were as follows: IL‐34, 5′‐TTGACGCAGAATGAGGAGTG‐3′ (forward); 5′‐CCCTCGTAAGGCACACTGAT‐3′ (reverse); GAPDH, 5′‐CAGGAGGCATTGCTGATGAT‐3′ (forward); 5′‐GAAGGCTGGGGCTCATTT‐3′ (reverse).

### 2.9 Statistical analysis

All statistical analyses were performed using SPSS Software 20.0 (SPSS, Inc). Results are given as means ± standard deviation. *t* Test was used to compare the differences between two groups. Spearman's correlation analysis was performed to evaluate the relationship between IL‐34 and clinicopathological features. GraphPad Prism 5 software was used to analyze data and create graphs. *P* values < .05 were considered statistically significant.

## 3 RESULTS

### 3.1 Serum IL‐34 is increased in patients with PTC and is associated with clinicopathological features

**FIGURE 1:**
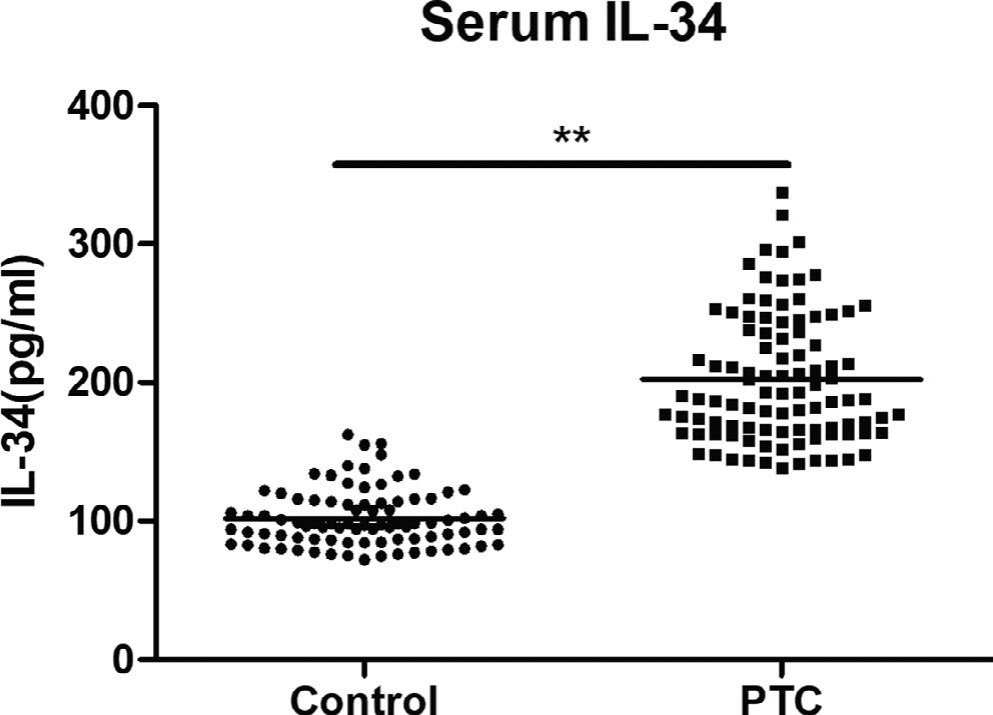
Serum IL‐34 expression in patients with PTC and controls. Dot plots showing the serum levels of IL‐34 in patients with PTC and controls. IL‐34 concentrations were analyzed by ELISA. **P < .01 was considered significant

IL‐34 expression was examined in 99 PTC patients and 87 age‐gender matched controls. As displayed in Figure 1, the serum levels of IL‐34 were significantly increased in PTC patients (202.46 ± 46.26 pg/mL) compared with those in controls (102.20 ± 20.41 pg/mL, *P* < .05). As shown in Table 1, the overexpression of IL‐34 was significantly associated with tumor size (*P* < .05), tumor TNM stage (*P* < .05), and lymph node metastasis (*P* < .05). No correlations were found with gender and patient age. These findings indicate that serum IL‐34 levels are higher in patients with PTC compared with matched controls and are correlated with PTC stage and lymph node metastasis.

**TABLE 1:** Correlations between IL‐34 levels and clinicopathologic features of 99 thyroid carcinomas patients

Characteristics	No. of patients (%)	*P* value
Gender		
Male	29 (29.3)	.865
Female	70 (70.7)	
Age (y)		
<45	54 (54.5)	.412
≥45	45 (45.5)	
LNM		
Yes	73 (73.7)	<.001*
No	26 (26.3)	
TNM stage		
I	47 (47.5)	<.001*
II	28 (28.3)	
III	15 (15.2)	
IV	9 (9)	
Tumor size (cm)		
<2	63 (64)	<.001*
≥2	18 (36)	

Abbreviations: LNM, lymph node metastasis; TNM, tumor node metastasis.

* *P *< .05 was considered significant.

### 3.2 IL‐34 expression is highly increased in PTC tissues

**FIGURE 2:**
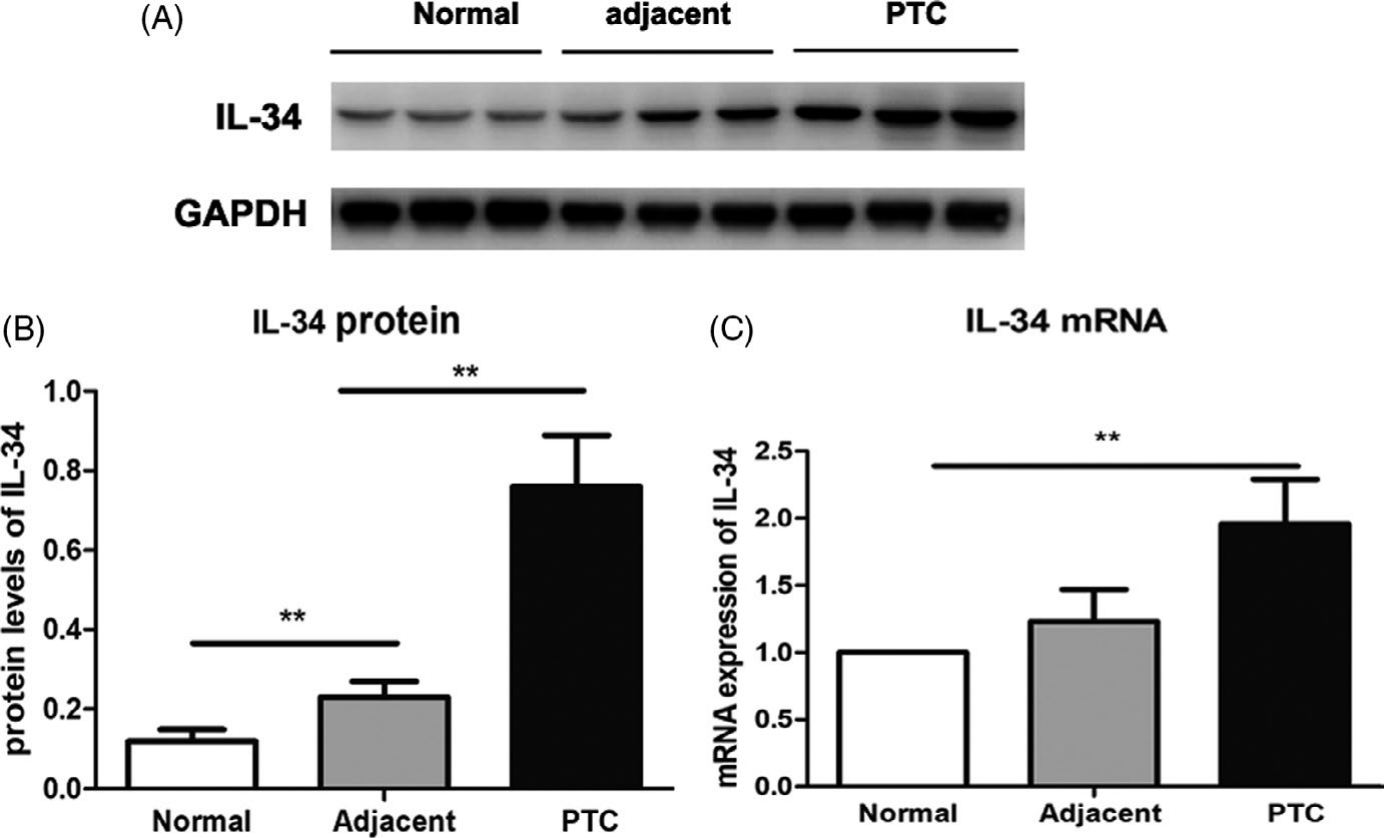
Protein expression of IL‐34 from human PTC samples. A, Western blot analysis of IL‐34 expression in PTC tissues, their adjacent noncancerous tissues, and normal controls. B, The ratio of IL‐34/GAPDH was determined to give a mean net density. C, Real‐time PCR analysis of IL‐34 expression in PTC tissues, their adjacent noncancerous tissues, and normal controls. Data were presented as the means ± standard error. **P < .01 was considered significant

To further determine the expression pattern of IL‐34 in human PTC tissues, we used Western blot assay to compare the expression levels of IL‐34 in 16 pairs of PTC tissues and matched adjacent tissues. We also collected the samples from normal subjects who subjected to thyroid nodules. The results showed that the mRNA and protein expression of IL‐34 was significantly higher in PTC tissues than in normal and adjacent tissues (*P* < .05, Figure 2A‐C).

### 3.3 IL‐34 promotes proliferation on PTC cells

**FIGURE 3:**
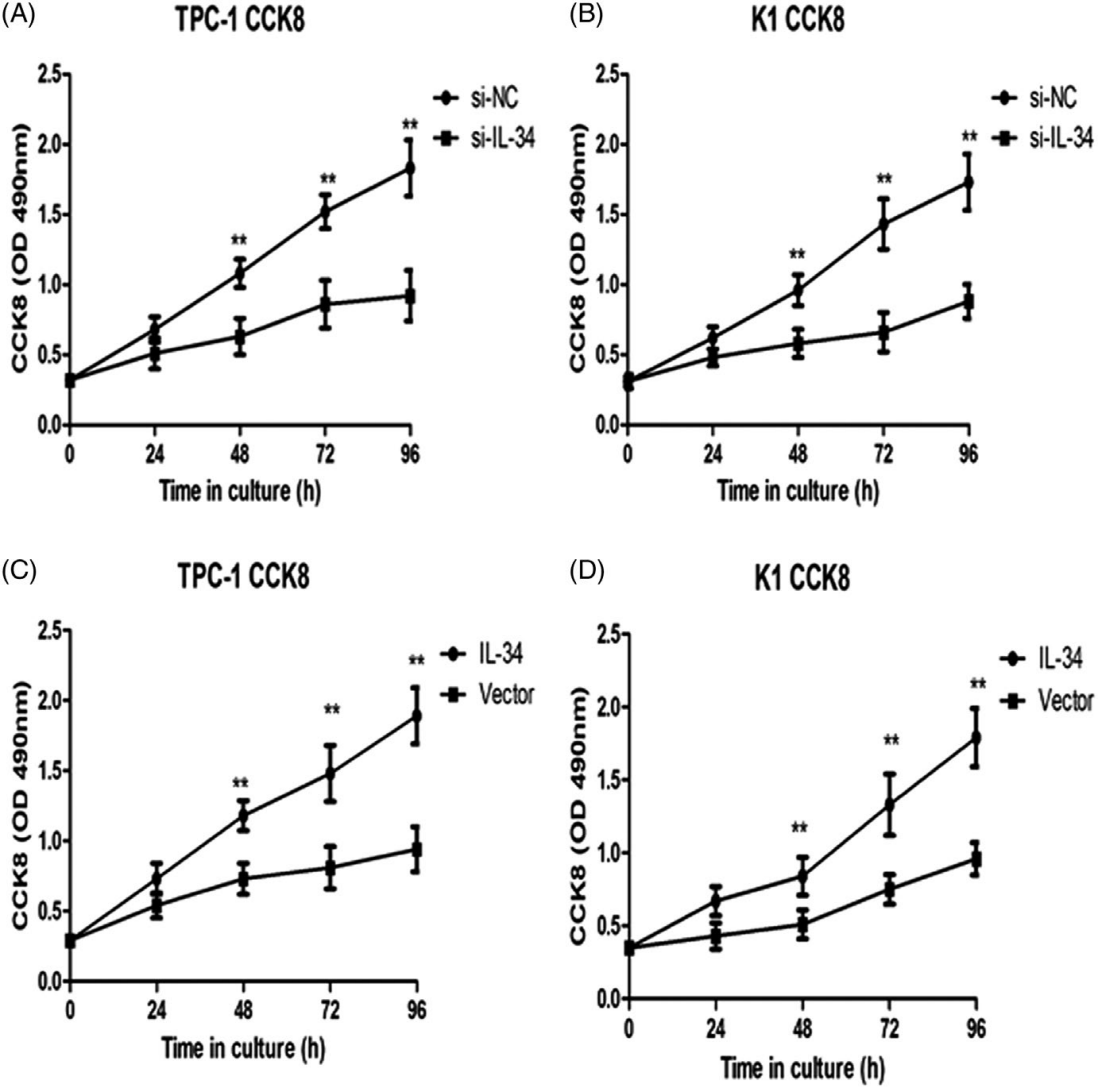
IL‐34 promotes proliferation on PTC cells. Cell proliferation was determined via the CCK‐8 assay to examine the proliferation of TPC‐1 (A) and K1 cells (B) in accordance to transfected with si‐NC or si‐IL‐34. Cell proliferation in TPC‐1 (C) and K1 cells (D) which were infected with lenti‐vector or lenti‐IL‐34. Data were presented as the means ± standard error. **P < .01 was considered significant

To evaluate the effect of IL‐34 on PTC cell proliferation, two cell lines (TPC‐1 and K1) were transfected with si‐IL‐34 or si‐NC for 48 hours and CCK‐8 assay was performed. As shown in Figure 3A,B, RNAi‐induced knockdown of IL‐34 led to a significant reduction in TPC‐1 and K1 cell proliferation compared with controls (*P* < .01, *P* < .01, respectively). As shown in Figure 3C,D, the CCK8 assay confirmed that IL‐34 overexpression significantly promoted TPC‐1 and K1 cell proliferation (*P* < .01, *P* < .01, respectively). This result suggested that IL‐34 plays a vital role in PTC proliferation.

### 3.4 IL‐34 inhibits apoptotic rates on PTC cells

**FIGURE 4:**
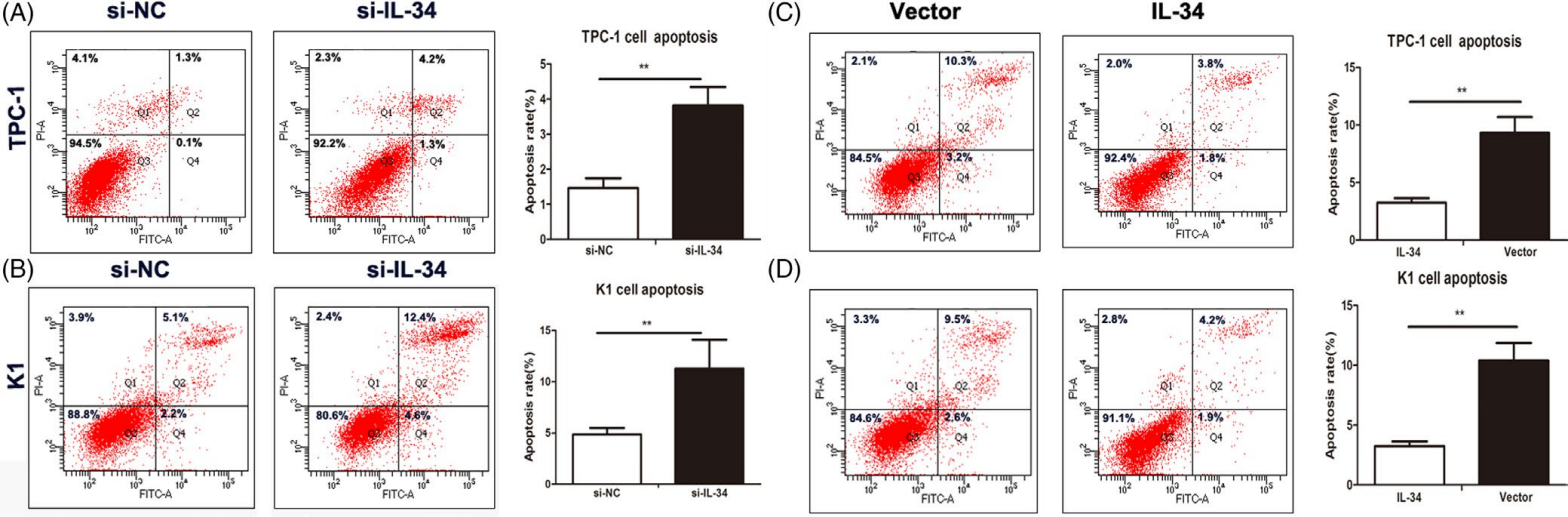
IL‐34 inhibits apoptotic rates on PTC cells. Cell apoptosis was determined by flow cytometry after transfected with si‐IL‐34 or si‐NC in TPC‐1 (A) and K1 cells (B). Cell apoptosis in TPC‐1 (C) and K1 cells (D) which were infected with lenti‐vector or lenti‐IL‐34. The apoptotic rates were shown. Data were presented as the means ± standard error. **P < .01 was considered significant

We performed flow cytometry analysis to study the effect of IL‐34 knockdown on apoptotic rates of two PTC cell lines. The results shown that knockdown of IL‐34 increased the apoptotic rates in TPC‐1 and K1 cell lines (*P* < .01, *P* < .01, respectively, Figure 4A,B). In contrast, we found the cell apoptotic rates of TPC‐1 and K1 cells were significantly suppressed by IL‐34 overexpression (*P* < .01, *P* < .01, respectively, Figure 4C,D).

### 3.5 IL‐34 promotes the invasion via on PTC cells

**FIGURE 5:**
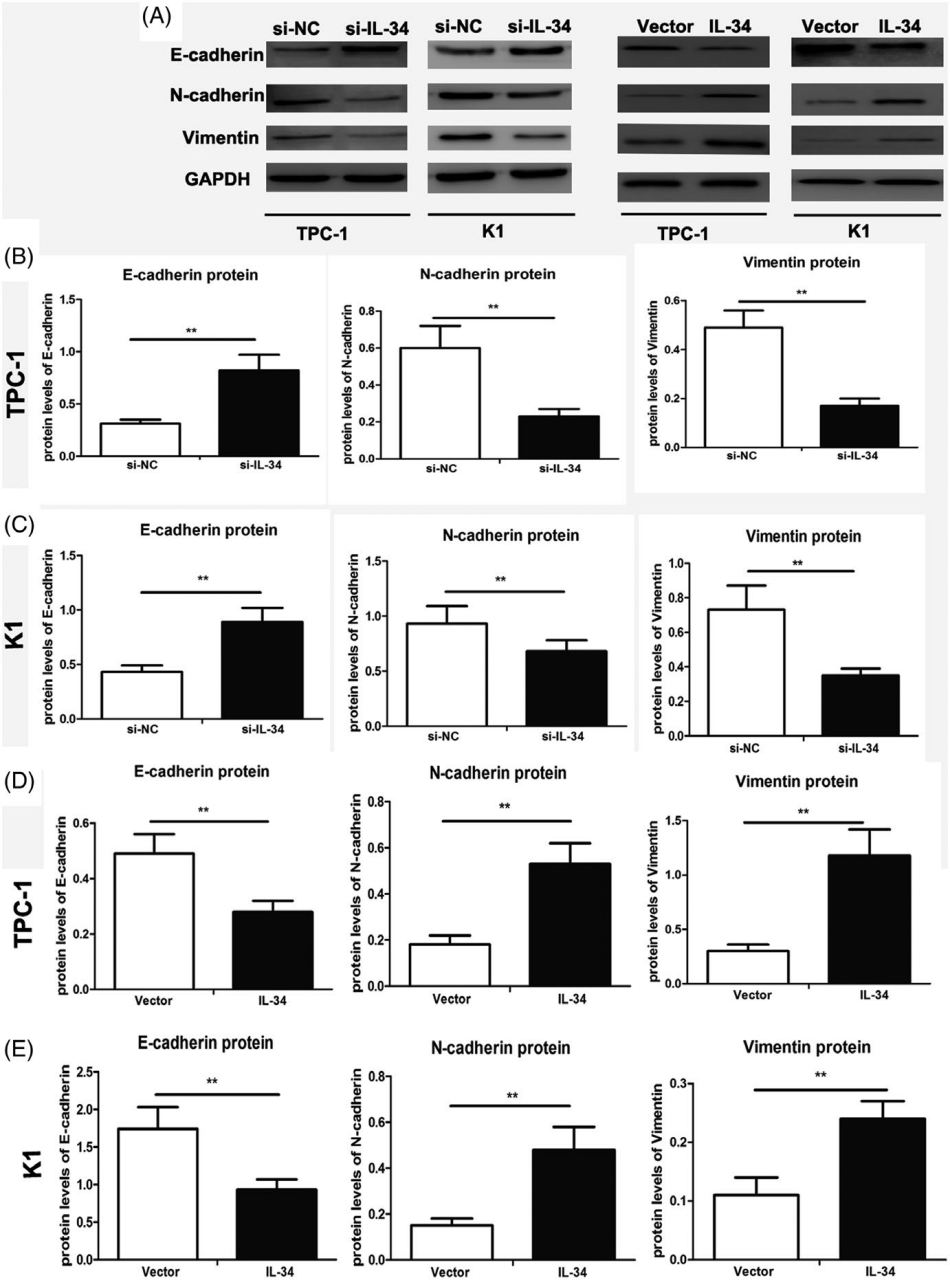
IL‐34 promotes the invasion via on PTC cells. Two PTC cell lines were not only transfected with si‐IL‐34 or si‐NC were also infected with lenti‐vector or lenti‐IL‐34 for 48 h. A, The protein expression levels of E‐cadherin, N‐cadherin, and Vimentin were measured by Western blotting in PTC cells. The ratios of EMT biomarkers when transfected with si‐IL‐34 or si‐NC were determined to give a mean net density in TPC‐1 (B) and K1 cells (C). The ratios of EMT biomarkers when infected with lenti‐vector or lenti‐IL‐34 were determined to give a mean net density in TPC‐1 (D) and K1 cells (E). Data were presented as the means ± standard error. **P < .01 was considered significant

EMT is a crucial process in carcinoma progression. We evaluated the effect of IL‐34 on EMT in PTC cells by detecting expression of the EMT biomarkers E‐cadherin, N‐cadherin, and vimentin. Western blot assay showed that the protein expression of E‐cadherin was significantly increased after IL‐34 knockdown in TPC‐1 cells compared with si‐NC cells, while the expression of N‐cadherin and vimentin was notably reduced (*P* < .01, *P* < .01, *P* < .01, respectively, Figure 5A,B). In addition, as demonstrated in Figure 5A,C, knockdown of IL‐34 markedly increased the expression of E‐cadherin in K1 cells, but reduced the expression of N‐cadherin and vimentin (*P* < .01, *P* < .01,* P* < .01, respectively). Conversely, we found that overexpression of IL‐34 promoted the carcinoma progression via EMT biomarkers (*P* < .01, *P* < .01, *P* < .01, respectively, Figure 5A,D,E). Taken together, these data show that IL‐34 participates in tumor progression via the EMT phenotype in PTC cells.

### 3.6 IL‐34 promotes the invasion and activates ERK signaling pathway on PTC cells

**FIGURE 6:**
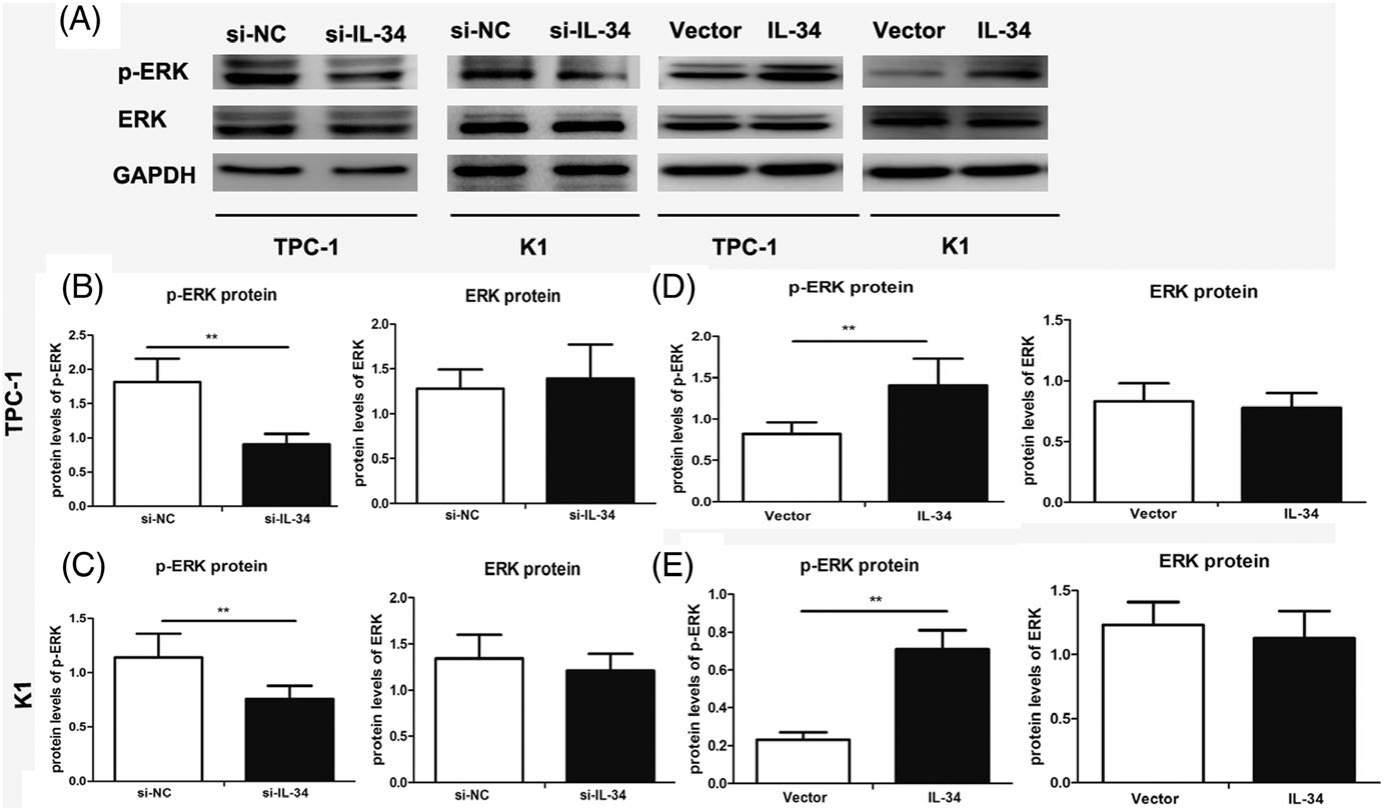
IL‐34 promotes the invasion and activates the ERK signaling pathway on PTC cells. Two PTC cell lines were transfected with si‐IL‐34 or si‐NC were also infected with lenti‐vector or lenti‐IL‐34 for 48 h. A, The protein expression levels of p‐ERK and ERK were measured by Western blotting in PTC cells. The ratios of ERK and p‐ERK when transfected with si‐IL‐34 or si‐NC were determined to give a mean net density in TPC‐1 (B) and K1 cells (C). The ratios of ERK and p‐ERK when infected with lenti‐vector or lenti‐IL‐34 were determined to give a mean net density inTPC‐1 (D) and K1 cells (E). Data were presented as the means ± standard error. **P < .01 was considered significant

The ERK signaling pathway plays an important role in tumor progression, including PTC cells. To determine whether IL‐34 influences activation of ERK signaling, we analyzed the protein levels of p‐ERK and t‐ERK in PTC cells by Western blot assay. As shown in Figure 6A,B, knockdown of IL‐34 significantly reduced the protein levels of p‐ERK in TPC‐1 cells compared with si‐NC transfected cells (*P* < .01). Moreover, we also found a significant reduction of p‐ERK in si‐IL‐34 transfected K1 cells compared with the si‐NC group (*P* < .01, Figures 6A and 5C). There was no significant change in t‐ERK protein expression level in the two PTC cell lines examined. Interestingly, we found that the phosphorylation of ERK was significantly increased through the overexpression of IL‐34, while the total ERK expression remains with no differences (*P* < .01, Figure 6A,D,E).

## 4 DISCUSSION

The present study found that IL‐34 expression was significantly increased in the serum and tissue samples of patients with PTC and was associated with clinicopathological characteristics. The upregulation of IL‐34 was significantly associated with tumor size, tumor node metastasis (TNM) stage, and lymph node metastasis (LNM). Furthermore, we also demonstrated that IL‐34 might promote the proliferation and suppress apoptosis in two PTC cell lines. Moreover, IL‐34 promotes the EMT phenotype and activates ERK signaling pathway on PTC cells**.** These findings suggest that IL‐34 might serve as a novel diagnostic biomarker in PTC.

IL‐34 has been demonstrated to mediate multiple cellular processes including cell proliferation and growth, cell cycle progression, and protein phosphorylation.^12^ It has been reported that IL‐34 contributes to the activation and progression of multiple cancers and is closely related to mortality in brain and lung cancers.^25^, ^26^ Interestingly, IL‐34 was found to be highly increased in patients with advanced stages of lung cancer compared with early stages.^27^ IL‐34 has been shown to be over‐secreted in an in vitro study of breast cancer.^22^ Moreover, IL‐34 expression was highly increased in patients with hepatocellular carcinoma and was correlated with poor prognosis and survival rates.^24^ To the best of our knowledge, ours is the first study to report that patients with PTC show elevated expression of IL‐34. Our results are consistent with previous findings in other cancer types. We found that expression of IL‐34 was significantly upregulated in tissue samples from patients with PTC. In addition, we found that IL‐34 is remarkably increased in the serum of patients with PTC and that the levels are closely related to pathological characteristics including tumor size and TNM stage. Moreover, knockdown of IL‐34 markedly inhibited the proliferation of PTC cells. Reversely, overexpression of IL‐34 significantly promotes the proliferation. These results indicate that IL‐34 may function as an oncogene in the metastasis of PTC.

We further established that IL‐34 might promote the proliferation and suppress apoptosis. An earlier report demonstrated that the apoptotic rates in thyroid cells were markedly reduced with IL‐34 stimulation.^28^ These results suggest that IL‐34 contributes to the proliferation of PTC cells by influencing apoptosis.

EMT is a process that contributes to the invasion and metastasis of cancer, including in PTC cells.^29^, ^30^ Reduced expression of the epithelial marker E‐cadherin and elevated expression of the mesenchymal marker N‐cadherin are principal characteristics of EMT.^31^ Previous studies found that E‐cadherin deficiency was related to higher TNM stage and increased degree of lymph node metastasis in PTC.^32^, ^33^ In addition, Baud'Huin et al reported that silencing IL‐34 may inhibit cell invasion and metastasis in cancer‐induced osteoclastogenesis.^17^ Our results are in accordance with previous studies and demonstrate that IL‐34 participate in tumor progression via the EMT phenotype in PTC cells. These results indicate that IL‐34 contributes to tumor metastasis by activating EMT in PTC cells.

We also studied the potential pathway through which IL‐34 regulates PTC progression. The ERK signaling pathway plays an important role in various cancers. Moreover, ERK activation is involved in tumor metastasis via multiple mechanisms, such as the regulation of cell invasion and EMT progression.^34^ Previous studies have reported that activation of the ERK signaling pathway promotes EMT progression in lung, pancreatic, and hepatocellular carcinoma.^35^, ^36^, ^37^ Franzè et al reported that IL‐34 activates ERK signaling in colorectal cancer. Moreover, inhibiting ERK signaling abolished the effect of IL‐34 on tumor progression.^38^ Our results are consistent with these earlier reports, suggesting that IL‐34 promotes the invasion and activates the ERK signaling pathway on PTC cells.

In conclusion, our study provides novel evidence that IL‐34 contributes to PTC progression. IL‐34 might promote the proliferation, EMT phenotype, and the ERK signaling pathway and suppress apoptosis. Therefore, IL‐34 may be an effective target for the therapy of PTC, and further studies are warranted to investigate its effects in vivo.

## CONFLICT OF INTEREST

The authors declare that they have no conflict of interest.
